# How we treat esophageal squamous cell carcinoma

**DOI:** 10.1016/j.esmoop.2023.100789

**Published:** 2023-02-13

**Authors:** H.C. Puhr, G.W. Prager, A. Ilhan-Mutlu

**Affiliations:** Department of Medicine I, Division of Oncology, Medical University of Vienna, Vienna, Austria

**Keywords:** cancer, esophageal, squamous cell carcinoma, treatment

## Abstract

Esophageal squamous cell carcinoma (ESCC) poses a major challenge for clinicians as the prognosis is poor and treatment options are limited. However, recent advances in immunotherapy have significantly changed the treatment algorithm of ESCC. Patients with early ESCC should undergo an endoscopic resection. If histological margins are infiltrated with tumor cells or other risk factors for lymph node metastasis are present, further resective surgery should be offered. In a locally advanced setting, radiochemotherapy with or without resection remains the standard of care. In the absence of pathological complete response after neoadjuvant radiochemotherapy and R0 resection, adjuvant immunotherapy for 1 year should be administered to improve disease-free survival. In metastatic first-line setting, combination of platin/fluoropyrimidine-based systemic chemotherapy with checkpoint inhibitors is the novel standard of care for all-comers in the United States and for patients with programmed death-ligand 1 positivity in Europe. Immunotherapy has also been approved in a second-line setting. However, the benefit from immunotherapy reinduction is still unknown and, therefore, standard second-line chemotherapy with taxanes or irinotecan is still the treatment of choice after progression on immunochemotherapy. It is of highest importance that treatment decisions are based on informed patient wishes and are discussed in an interdisciplinary tumor board. This review summarizes how to manage, in our opinion, patients with ESCC and gives a practical overview of the treatment strategies in Europe.

## Highlights

• Novel immunotherapeutic agents have changed the way we treat ESCC.
• Locally advanced ESCC is treated with radiochemotherapy with or without surgery depending on localization and stage.
• In absence of complete pathological response after radiochemotherapy and R0 resection adjuvant immunotherapy is indicated.
• Tumor proportion score (TPS) and combined positive score (CPS) should be evaluated in advanced disease.
• As approved in Europe, patients with advanced TPS ≥1% or CPS ≥10 ESCC should be treated with first-line immunochemotherapy.

## Introduction

Esophageal cancer represents a global health issue with over 600 000 newly diagnosed cases per year, which incorporates >3% of all cancer cases.^1^ While esophageal squamous cell carcinoma (ESCC) is still the most prevalent subtype worldwide, an increase in esophageal adenocarcinoma cases has been registered, especially in more developed countries. While changing epidemiology provides novel challenges, recent advances have also altered our perspective on how to treat esophageal cancer.

However, choosing therapeutic strategies for patients represents a major challenge for clinicians as prognosis remains poor, especially in advanced settings. Thus, it is of highest importance to provide physicians with adequate guided strategies.

**Figure 1:**
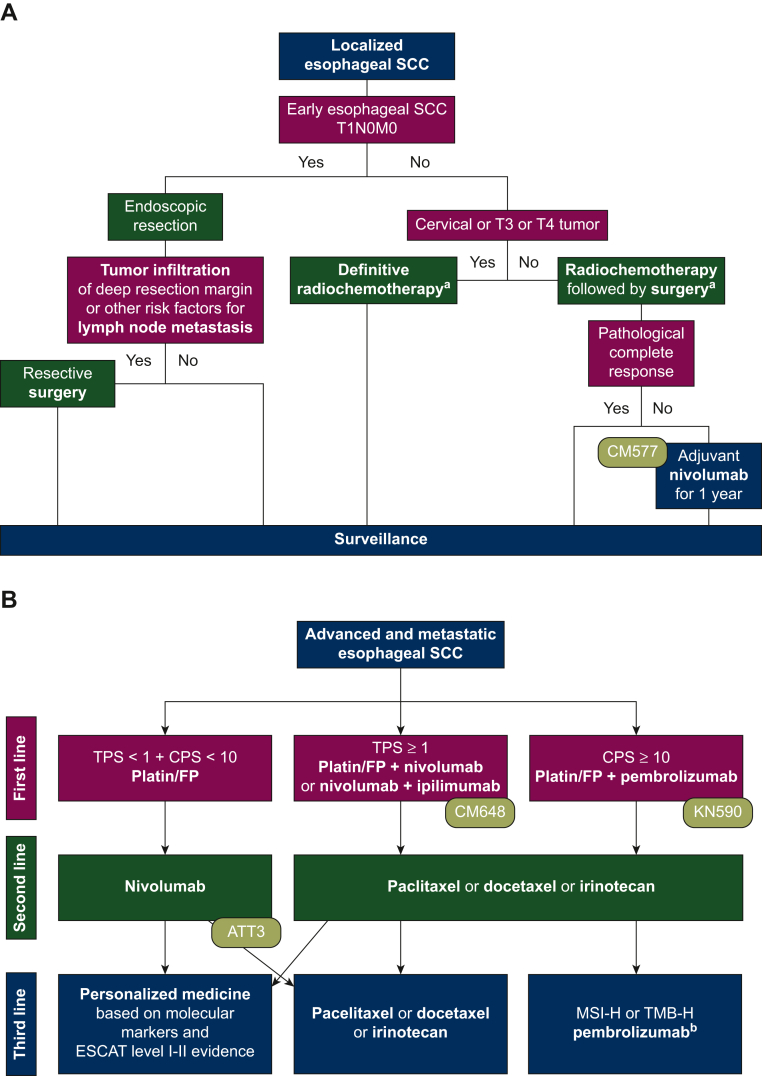
**Algorithm for the treatment of locally advanced (A) and advanced (B) esophageal SCC.** ATT, ATTRACTION; CM, CheckMate; CPS, combined positive score; ESCAT, ESMO Scale for Clinical Actionability of molecular Targets; FP, fluoropyrimidine; KN, KEYNOTE; SCC, squamous cell carcinoma; TPS, tumor proportion score. ^a^40-55 Gy in resectable setting, up to 65 Gy in definitive setting. Chemotherapy with platin/taxane and platin/fluoropyrimidine available. ^b^Not approved by European Medicines Agency but by United States Food and Drug Administration.

This article focuses on our opinion on the treatment of ESCC based on molecular markers as well as current approval status of European and American authorities. As the treatment approaches significantly differ among geographic locations, this review will primarily reflect the European standards, and will shed some light on the American and Asian perspectives. Despite varying treatment labels among different countries, providing timely and equitable access to novel cancer medicines remains one of the main pillars of quality cancer care worldwide. Figure 1 summarizes the treatment strategies discussed in this article.

## Diagnosis, staging and treatment planning

Patients with esophageal cancer often present with major symptoms such as dysphagia, gastrointestinal bleeding, recurrent aspiration or emesis and weight loss.^2^ Especially squamous cell carcinomas, which are usually located in the proximal to middle esophagus, may lead to a fast deterioration of the patient’s physical status due to insufficient nutrition intake. Thus, patients experiencing those difficulties should undergo an upper intestinal endoscopy. Six to eight representative biopsies should be taken from all suspicious areas, respectively. The histological tumor type should then be classified according to the World Health Organization criteria.

Further staging should be done according to the current TNM (tumor–node–metastasis) classification and includes complete clinical examination and computed tomography (CT) or positron emission tomography–CT of the neck, thorax and abdomen. In addition, the use of endoscopic ultrasound and bronchoscopy for assessment of T status with invasion toward the surrounding organs and for identification and biopsy of suspected lymph node metastases outside the regular local treatment limits is optional but not mandatory.

Due to the complexity of patient management and poor prognosis, multidisciplinary assessment and planning of treatment are mandatory. Supportive care such as nutritional and physical therapy, psychosocial support as well as palliative care should be recommended to every patient.^3^

### Molecular markers

Immunohistochemical analysis of programmed death-ligand 1 (PD-L1) is recommended in advanced settings in order to decide eligibility for immunotherapy. The tumor proportion score (TPS) represents viable tumor cells with partial or complete membrane staining at any intensity, while the combined positive score (CPS) also includes positive lymphocytes and macrophages.^3^ Although recent analyses showed that results can vary depending on the immunohistochemistry assay,^4^ PD-L1 remains an essential predictive marker. It is important to note that approvals for immunotherapeutic drugs are strictly linked to the PD-L1 scoring methodology used in each landmark trial, but not linked to any specific immunohistochemistry method or PD-L1 antibody clone for testing. Trials on the interchangeability of the existing assays in squamous cell carcinoma (SCC) patients are ongoing.

Further molecular markers, which are associated with response to immunotherapy, are microsatellite instability high (MSI-H), mismatch repair (MMR) deficient and tumor mutational burden high (TMB-H) tumors. The evaluation of these markers in ESCC is not mentioned in current guidelines;^3^ however, as these molecular subtypes showed excellent response to immunotherapy, a so-called ‘tissue agnostic approval’ of pembrolizumab was granted for advanced tumors without other satisfactory alternative treatment options by the United States Food and Drug Administration (FDA).^5^^,^^6^ Thus, the evaluation of MSI, MMR and TMB, although rarely detected in ESCC, should be considered in advanced stages without other treatment options.

## Localized setting

### Early disease

Patients with an early ESCC staged as T1N0M0 should undergo an endoscopic en bloc resection, using preferably endoscopic submucosal dissection or endoscopic mucosal resection. If the deep resection margin is infiltrated by tumor cells or the patients show risk factors for lymph node metastases such as lymphovascular invasion, low differentiation grade, ulceration and large tumor size, further resective surgery with lymphadenectomy is recommended.^3^

### Locally advanced setting

ESCCs are considered locally advanced when staged T2-4 or N1-3 with M0. The therapeutic regimen in localized settings is independent of molecular markers and comprises radiochemotherapy with or without resection (Figure 1A). Recently published 10-year overall survival (OS) data of the CROSS trial showed that especially patients with ESCC (*n* = 84) profit from the addition of radiochemotherapy (carboplatin + paclitaxel + radiation therapy with 41.4 Gy in 23 fractions) compared to surgery alone (46% versus 23%, *P* = 0.007, respectively).^7^

However, risk of recurrence remains high despite multimodal treatment strategies. The CheckMate 577 trial showed that the addition of adjuvant nivolumab for 1 year improves the disease-free survival in patients (ESCC *n* = 230) with R0 resections and residual pathological disease compared to placebo [22.4 versus 11.0 months; hazard ratio (HR) 0.69; *P* < 0.001].^8^ These results led to a paradigm change and the approval of adjuvant nivolumab for patients without complete pathological response after radiochemotherapy and R0 resection by the European Medicines Agency (EMA) and FDA.

A *post hoc* analysis showed that especially patients with a baseline CPS ≥5 had the most benefit, yet the positive effect could also be seen in CPS <5 patients.

However, surgery is not the optimal treatment for every patient. Due to high perioperative morbidity in cervical and locally advanced (T3-T4) ESCC, definitive radiochemotherapy with up to 65 Gy and either a platin/taxane (carboplatin + paclitaxel, CROSS regimen) or a platin/fluoropyrimidine-based (5-fluorouracil with cisplatin or oxaliplatin, PRODIGE5/ACCORD17 regimen) chemotherapy is the standard of care in this patient cohort.^9^^,^^10^ Whether these patients also profit from immunotherapy is currently under investigation in several clinical trials.

## Advanced/metastatic setting

Patients are characterized as advanced when the cancer is metastatic or cannot be resected or treated with definitive radiochemotherapy. Current treatment algorithms and more data on recent paradigm-changing trials are shown in Figure 1B and Table 1.

**Table 1:** Clinical trials providing novel treatment options for metastatic ESCC patients

Clinical trial	Treatment	OS of ESCC patients PD-L1 independent				OS of ESCC patients PD-L1 dependent			
		*n*	OS in months	OS HR	OS *P* value	*n*	OS in months	OS HR	OS *P* value
KEYNOTE-590	• Cisplatin/fluoropyrimidine + pembrolizumab • Cisplatin/fluoropyrimidine + placebo	548 (274 versus 274)	12.4 versus 9.8	0.73 (95% CI 0.62-0.86)	<0.0001	CPS ≥ 10 286 (143 versus 143)	13.9 versus 8.8	0.57 (95% CI 0.43-0.75	<0.0001
CheckMate 648	• Nivolumab + chemotherapy • Cisplatin/fluoropyrimidine	645 (321 versus 324)	13.2 versus 10.7	0.74 (99.1% CI 0.58-0.96)	0.002	TPS ≥ 1 315 (158 versus 157)	15.4 versus 9.1	0.54 (99.5% CI 0.37-0.80)	<0.001
CheckMate 648	• Nivolumab + ipilimumab • Cisplatin/fluoropyrimidine	649 (325 versus 324)	12.7 versus 10.7	0.78 (98.2% CI 0.62-0.9)	0.01	TPS ≥ 1 315 (158 versus 157)	13.7 versus 9.1	0.64 (98.6% CI 0.46-0.90)	0.001
ATTRACTION-3	• Nivolumab • Paclitaxel or docetaxel	419 (210 versus 209)	10.9 versus 8.4	0.77 (95% CI 0.62-0.96)	0.019	n.a.			
KEYNOTE-181	• Pembrolizumab • Chemotherapy (paclitaxel, docetaxel or irinotecan)	401 (198 versus 203)	8.2 versus 7.1	0.78 (95% CI 0.63-0.96)	0.0095	CPS ≥ 10 249 (167 versus 82)	10.3 versus 6.7	0.64 (95% CI 0.46-0.90)	n.a.

CI, confidence interval; CPS, combined positive score; ESCC, esophageal squamous cell carcinoma; HR, hazard ratio; *n*, number of patients; n.a., not available; OS, overall survival; PD-L1, programmed death-ligand 1; TPS, tumor proportion score.

### First-line therapy

In the first-line setting, platin/fluoropyrimidine-based chemotherapy with or without checkpoint inhibitors presents the current treatment standard.

In the double-blind KEYNOTE-590 trial, the combination of chemotherapy (cisplatin + 5-fluorouracil) and pembrolizumab showed a significant survival benefit. A subgroup analysis of the SCC population (*n* = 548) shows that more than half of the patients had a CPS ≥10 [*n* = 286 (52%)]. Especially in this subgroup the addition of pembrolizumab led to a major benefit (HR 0.57; *P* < 0.0001).^11^

In addition, the CheckMate 648 trial randomized patients into three arms: (i) nivolumab + chemotherapy (cisplatin + 5-fluorouracil), (ii) nivolumab + ipilimumab or (iii) chemotherapy (cisplatin + 5-fluorouracil) alone. Nivolumab + chemotherapy led to a significant survival benefit for the whole population, yet especially for patients with TPS ≥1% (HR 0.54; *P* < 0.001). Furthermore, also the dual inhibition with nivolumab + ipilimumab showed a significant OS benefit for patients with TPS ≥1% (HR 0.64; *P* = 0.001).^12^ Although the dual checkpoint inhibition showed less major adverse events and slightly higher objective response rates in TPS ≥1% patients, the survival curves raise concern as they cross each other a few months after therapy initiation indicating a higher mortality for patients receiving nivolumab + ipilimumab with respect to chemotherapy alone. In addition, progression-free survival values in this arm were non-superior when compared to chemotherapy alone.^12^

Based on these results, combination therapies with either pembrolizumab + chemotherapy, nivolumab + chemotherapy or nivolumab + ipilimumab are possible options for the first-line treatment of advanced ESCC. Although the FDA approved these regimens independent of PD-L1 expression, the EMA was more restrictive choosing to approve the pembrolizumab only for CPS ≥10 and nivolumab or nivolumab + ipilimumab only for TPS ≥1% patients. Both agencies have not restricted the type of chemotherapy backbone, allowing both oxaliplatin and cisplatin to be combined with capecitabine and 5-fluorouracil. However, due to the aforementioned concerns about the data on dual checkpoint inhibition, the nivolumab + ipilimumab regimen has a lower grade of recommendation in clinical practice when compared to nivolumab + chemotherapy.

Chemotherapy combined with either nivolumab or pembrolizumab presumably provides equal outcome, thus the physician is free to decide on one of the aforementioned substances. Treatment decisions may then be based on the administration interval. Although the approvals were not restricted to specific platin/fluoropyrimidine backbones, nivolumab can be administered every 2 weeks being more feasible with oxaliplatin + 5-fluoruracil and pembrolizumab every 3 weeks with cisplatin + 5-fluoruracil and capecitabine-based regimen. For patients with TPS <1% and CPS <10, currently platin/fluoropyrimidine-based chemotherapy remains the standard of care in Europe.^3^

Further immune checkpoint inhibitor drugs [ESCORT-1 (camrelizumab),^13^ JUPITER-06 (toripalimab)^14^ and ORIENT-15 (sintilimab)^15^] showing benefit for survival in combination with either taxane- or platin-based chemotherapy in entirely Asian patients yet have no approval from FDA or EMA. Tislelizumab (RATIONALE-306) demonstrated benefit both in Asian and Caucasian populations, thus wider approvals are awaited.^16^

### Further-line therapies

Taxanes (paclitaxel or docetaxel) or irinotecan monotherapies are recommended as further-line treatment options.^17^ However, the Asian ATTRACTION-3 study compared taxane-based chemotherapy to nivolumab, which significantly improved median OS^18^ and, thus, was approved by EMA and FDA independent of PD-L1 status.

Another approval by the FDA was based on the KEYNOTE-181 trial, which showed a survival benefit for patients with CPS ≥10 esophageal cancer receiving pembrolizumab over standard second-line chemotherapy in a second-line setting.^19^ However, as the study was not originally designed to test the superiority in this subgroup, resulting in major imbalances in the distribution of patient characteristics and impact on the interpretation of the results, the EMA did not approve this regimen.

It is important to note that both trials included patients after failure of standard first-line treatment of that time (platin-based or fluoropyrimidine-based chemotherapy) and there were no patients with prior checkpoint inhibition within the patient cohorts. Whether immunotherapy reinduction is feasible still has to be addressed in future analyses. Thus, chemotherapy is still recommended after progression on combination therapy, making second-line immunotherapy only feasible and the new standard of care for patients who did not receive prior checkpoint inhibition.

As therapeutic options for further-line therapies are rare, molecular profiling should be considered early on. Pembrolizumab provides a feasible option for MSI-H and TMB-H tumors independent of localization and histology, yet those markers are scarce in ESCC. Further molecular analyses may provide further precision medicine approaches. Experimental approaches with a European Society for Medical Oncology (ESMO) Scale for Clinical Actionability of molecular Targets (ESCAT) level I or II are considered to provide the most beneficial outcome.^20^

Reinduction therapy of previously administered treatment strategies might also provide further options and should be discussed when no other lines are available.

## Conclusions

Recent years have changed the treatment strategies in ESCC, both in localized and advanced settings as immunotherapy showed promising trial results. However, prognosis in advanced disease remains poor and has to be further improved by clinical investigations. Patients with specific biomarker expressions might benefit more from addition of immunotherapy. Respective treatment recommendations from approval authorities and differences on treatment labels should therefore be followed carefully. Therapeutic decisions should always be made by an interdisciplinary tumor board and in accordance with the patient’s wishes.
